# Evaluation of Stature Development During Childhood and Adolescence in Individuals with Familial Hypophosphatemic Rickets

**DOI:** 10.1100/tsw.2005.102

**Published:** 2005-10-17

**Authors:** Mauro M.S. Borghi, Veronica Coates, Hatim A. Omar

**Affiliations:** ^1^ Adolescent Clinic and Bone Metabolism Unit, Department of Pediatrics, Irmandade da Santa Casa de Misericórdia de São Paulo, Santa Casa de São Paulo, Faculty of Medical Sciences, São Paulo, Brazil; ^2^ Section of Adolescent Medicine, Department of Pediatrics, University of Kentucky, Lexington, USA

**Keywords:** rickets, vitamin D resistant rickets, short stature, growth hormone, growth, Brazil, United States

## Abstract

This review was conducted to study the diagnosis, treatment, and growth progression in infants and adolescents with familial hypophosphatemic rickets. The bibliographic search was carried out utilizing the electronic databases MEDLINE, OVID, and LILACS and by direct research within the last 15 years using the keywords rickets, familial hypophosphatemia, vitamin D deficiency, stature growth, childhood, and adolescence. Article selection was done by comparing the evaluation of the growth in patients with familial hypophosphatemic rickets, including the variables that might affect them, for possible future therapeutic proposals. It is concluded that the most significant fact in the treatment of familial hypophosphatemic rickets in infancy was the magnitude of the final stature. The use of growth hormone can be helpful in these patients. However, research reporting treatments with the use of the growth hormone for rickets are controversial. The majority of the authors agree that treatment using vitamin D and phosphate enables some statural growth in cases of early diagnosis, reflecting a better prognosis.

## INTRODUCTION

Rickets has been recognized as a disease since the period of the Roman Empire. Soranus, in 100 BCE, related a larger number of bone deformities in the inferior limbs of Roman infants when they were compared with the Greeks. In the same period, Galeno described the classical bone deformities presented by rickets[1]. In 1650, Francis Glisson published the treaty "De Rachitide Sive Morbo Puerili, Qui Vulgo" describing the clinical characteristics of rickets as widening of the epiphysis, the rachitic rosary, the femoral bowing, and consecrated the disease's name[2,3,4]. In 1937, Albright et al.[5] related for the first time, in a well defined criteria, rickets as a resistance to vitamin D. They defined this type of rickets as a clinical entity distinct from the other kinds of rickets. In 1958, Winters et al.[6] analyzed the genealogic tree of patients with familial hypophosphatemic rickets and established that the transmission mechanism was an X-linked autosomal dominant disorder.

In 1968, Coates et al.[7] described three patients who were bearers of vitamin D–resistant rickets with hypophosphatemia and it was the first national description of its nomenclature. The term vitamin D–resistant rickets was used to denominate this disease in face of the absence of a clinical answer. With the elucidation of its pathophysiology, it was then called X-linked hypophosphatemic rickets or familial hypophosphatemic rickets, therefore taking into consideration the loss of phosphate at the kidney level[8].

## GENETIC INHERITANCE

Transmission is an X-linked autosomal dominant disorder (77%), however, sporadic cases are described in 23% of the patients, possibly caused by spontaneous mutations[9]. The disease is caused by a mutation in the regulator gene of phosphate with homology for the endopeptidases (PHEX), located in the X chromosome in the Xp position 22.1-22.2, causing a reduction in the phosphatonin inactivator protein 10. Usually, the reabsorption of phosphate takes place in the proximal kidney tubule and it is done by the sodium and phosphate transporter known as NPT2. In familial hypophosphatemic rickets, the mutation of the PHEX gene stops repressing phosphatonin expression that, once elevated, maintains NPT2 inactivation. Consequently, there is reabsorption inhibition of phosphate by the kidney and an increase in phosphaturia. It is believed that there is no relationship between the severity of the disease and the kind of location or mutation of the PHEX gene[11].

## DIAGNOSIS

The characteristic diagnosis of the disease in the infant usually starts between the 18th month and the 24th month following parturition, after they start walking, with the progressive bowing of the inferior limbs[12] with valgus laxity less frequent than varius laxity[13]. The bone deformities appear in consequence to the body weight support on the mineralized bones, which are more flexible[14,15]. The low stature is described as one of the clinical characteristics of familial hypophosphatemic rickets[16,17] and can be limited to the inferior limbs[18]. Patients with familial hypophosphatemic rickets do not present with muscle weakness or hypotonicity, characteristic of nutritional rickets caused by vitamin D deficiency. Females are more affected[18], however, the phenotypic aspects are more important in the males[17].

## LABORATORY INVESTIGATION

Very low serum levels of phosphorus with elevation of serum alkaline phosphatase levels and normal calcium suggest the diagnosis of hypophosphatemic rickets and kidney metabolism of phosphate should be evaluated by the rate of kidney phosphate reabsorption (TRP) that is calculated in samples of urine and blood, collected simultaneously through the following formula:

$$\%\ \text{TRP} = 1 - \frac{\text{Phosphorus urine X Serum creatinine}}{\text{Phosphorus serum X creatinine urine}}\ \text{X}\ 100$$

The normal rate of phosphate reabsorption is between 85 and 100%[15]. We use the Walton and Bijouet nomogram for the determination of the tubular kidney phosphate threshold and, therefore, the rate of tubular phosphate reabsorption[19]. When the relationship between the maximum threshold for tubular phosphate (TMP) and the rate of glomerular filtration (GFR) is lower than the value of phosphate, we prove the kidney is losing phosphate. We have a quantification of the parathyroid hormone (PTH) and metabolic circulation of the 25-hydroxyvitamin D and 1.25 dihydroxyvitamin D, which is important prior to the beginning of treatment. In patients with nontreated familial hypophosphatemic rickets, the serum concentration of 25-hydroxyvitamin D is normal and the concentration of 1.25 dihydroxyvitamin D is in the lower limit of the normality[20]. There is no relationship between the seriousness of phosphate reduction and low stature[18].

## RADIOLOGY

The changes are more evident in the extremities of the long bones and appear radiographically as a flattening in the area of the epiphysis and of the metaphysis with concave and irregular edges that result in a cupping configuration. The femur might present a bowing defect and, as the bowing progresses, the asymmetrical aspect of the cartilage of growth becomes more evident[21]. The radiographic characteristics are usually more serious in the inferior limbs, revealing a thin cortex and rough aspect. The cup form with widening and bone rarefaction is usually less outstanding than the one observed in the nutritional rickets[10].

## CLINICAL TREATMENT

The treatment is not curative and aims to improve growth, reduce the severity of the bone alterations, and increase physical activity, as well as reduce the severity of the bowing defect. Calcitriol in fractional doses of 0.25–1.5 µg daily (larger doses are rare), added to elementary phosphorus on an average of 2 g daily fractioned in six doses[8], is recommended. Two weeks after the beginning of the treatment evaluation of the serum levels of calcium, PTH and the urinary excretion of calcium and of creatinine is needed. In this manner, we can maintain an appropriate balance in the use of calcitriol and of the phosphate syrup. The excess of the calcitriol results in vitamin D intoxication, hypercalcemia, hypercalciuria, and kidney calcinosis.

## SURGICAL TREATMENT

The aim of the osteotomy is the complete correction of the deformity with one surgical procedure, restoring both the knees and ankle to the horizontal plane. The surgery only happens after metabolic stabilization[22].

## STATURE DEVELOPMENT

Stature growth is one of the aims of familial hypophosphatemic rickets treatment. Several therapeutic regimens have been proposed, such as the use of the phosphate and vitamin D isolate and stature gain associated to the use of growth hormone (GH). There is a variety of therapeutic regimens, where authors like Glorieux et al.[17] in 1972, Verge et al.[23] in 1991, and Makitie et al.[24] in 2003 obtained an increase of stature when they used phosphate and vitamin D. Others like Friedman et al.[25] in 1993 and Seikaly et al.[26] in 1994 did not obtain a significant growth stature. Saggese et al.[27] in 1995, Shaw et al.[28] in 1995, Baroncelli et al.[29] in 2001, and Welch[30] in 2001obtained an increase in stature when they associated GH to the treatment using phosphate and the vitamin D, while others like Seikaly et al.[31] in 1997 and Cameron et al.[32] in 1999 could not obtain treatment response. According to Patel et al.[33] in 1996, the use of the GH would reduce the use of phosphate and the risk of complications regarding nephrocalcinosis.

During puberty, stature growth is normal, independent of the treatment plan, because of the hormone mechanisms involved, as it has been described by Balsan and Tieder[34] in 1990. The analysis of the variables in relation to the age therapeutic initiation and duration of the treatment did not show a relationship between statures, as it was described by Stickler and Morgenten[35] in 1989 and Verge et al.[23] in 1991.

Friedman et al.[25] in 1993 and Makitie et al.[24] in 2003, when they evaluated the gain in stature with the use of the vitamin D and phosphate, reported that the best criteria for prediction of the final stature were the percentile of the stature before therapy and that patients treated earlier presented a tendency to reach familial stature.

## DISCUSSION

Osteometabolic diseases encompass an extensive group of illnesses that alter the bone metabolism, resulting in an increase or general reduction of bone matter, abnormal production of osteoid tissue, mineralization, and skeleton abnormal storage disturbances.

During infancy, metabolic diseases such as scorbuts, hyperparathyroidism, mucopolysacharidosis, osteogenesis imperfecta, and rickets can be found with the latter being the most frequent. Hypophosphatemic familial rickets is the most common type of hereditary rickets, and patients carrying this disease might have short stature as a consequence of metabolic alterations. The evaluation of the growth stature in patients with XLH (X-linked hypophosphatemic rickets) and the variables, age at diagnosis and the age at the beginning of treatment with vitamin D and phosphate, as well as the final stature, reinforce the need for an early diagnosis of the disease and, moreover, confirm the importance of an immediate treatment with medication.

## CONCLUSIONS

The majority of the authors reported that the decrease in stature was present in the diagnosis in patient bearers of familial hypophosphatemic rickets and that treatment using vitamin D and phosphate promoted stature growth. The use of the growth hormone may be beneficial for these patients, however, in some studies stature growth was controversial in relation to the aforementioned treatment. Variables, such as the age at the diagnosis, percentile of the stature at the time of diagnosis, and treatment implementation, might reflect in the final stature, thus demonstrating the importance of an early diagnosis.

## ACKNOWLEDGMENTS

We are grateful to the Support Center for Scientific Publications of Santa Casa de São Paulo, Faculty of Medical Sciences for the editorial assistance.

## BIOSKETCHES

**Mauro M.S. Borghi**, **MD**, Medical Assistant, Bone Metabolism Unit, Department of Pediatrics, Irmandade da Santa Casa de Misericórdia de São Paulo, Santa Casa de São Paulo, Faculty of Medical Sciences, São Paulo, Brazil. E-mail: mjpborghi@terra.com.br

**Veronica Coates**, **MD**, Professor, Adolescent Clinic and Bone Metabolism Unit, Department of Pediatrics, Irmandade da Santa Casa de Misericórdia de São Paulo, Santa Casa de São Paulo, Faculty of Medical Sciences, São Paulo, Brazil. E-mail: veronicacoates@attglobal.net

**Hatim Omar, MD,** Professor of Pediatrics and Obstetrics/Gynecology and Director of Adolescent Medicine and Young Parent programs at the Kentucky Clinic, University of Kentucky in Lexington. Dr. Omar has numerous publications in child health, adolescent health, medicine, obstetrics, and gynecology. E-mail: haomar2@uky.edu
